# Expression quantitative trait locus analysis for translational medicine

**DOI:** 10.1186/s13073-015-0186-7

**Published:** 2015-06-24

**Authors:** Greg Gibson, Joseph E Powell, Urko M Marigorta

**Affiliations:** Center for Integrative Genomics, School of Biology, Georgia Institute of Technology, Atlanta, GA 30332 USA; Centre for Neurogenetics and Statistical Genomics, Queensland Brain Institute, University of Queensland, St Lucia, Brisbane, QLD 4072 Australia; The Institute for Molecular Bioscience, University of Queensland, Brisbane, QLD 4072 Australia

## Abstract

Expression quantitative trait locus analysis has emerged as an important component of efforts to understand how genetic polymorphisms influence disease risk and is poised to make contributions to translational medicine. Here we review how expression quantitative trait locus analysis is aiding the identification of which gene(s) within regions of association are causal for a disease or phenotypic trait; the narrowing down of the cell types or regulators involved in the etiology of disease; the characterization of drivers and modifiers of cancer; and our understanding of how different environments and cellular contexts can modify gene expression. We also introduce the concept of transcriptional risk scores as a means of refining estimates of individual liability to disease based on targeted profiling of the transcripts that are regulated by polymorphisms jointly associated with disease and gene expression.

## The importance of expression quantitative trait loci

The recognition that the majority of genotypes associated with diseases localize to regulatory regions [1, 2] rather than to protein-coding regions has increased interest in the effect of polymorphisms on gene expression. Although it may have started as something of a high-profile academic curiosity 10 years ago [3, 4], expression quantitative trait locus (eQTL) analysis has become a key tool for the functional understanding of the results of genome-wide association studies (GWASs) [5]. Several thousand papers now refer to human eQTLs, loci that associate with transcript abundance at genome-wide significance levels. They help to explain risk for diseases as diverse as autoimmune, cardiovascular and metabolic diseases, as well as cancer, by focusing attention on causal genes within a defined interval. eQTLs suggest mechanisms by which polymorphisms may influence gene function as it relates to disease, particularly where they alter experimentally or bioinformatically defined sequence elements. In addition, they may have a role in the prediction of the onset or course of a disease.

Recent trends in the field include meta-analysis of ever-larger sample sizes to increase power, investigation of more and more tissues, and incorporation of chromatin measures to explore the mechanisms by which eQTLs act. In addition, analytical algorithm development has progressed as RNA-sequencing (RNA-seq) has displaced microarrays as the primary means of measurement of transcript abundance. We start this review by explaining some of the key concepts and resources for exploring eQTLs, but the major purpose is to highlight the implications of eQTL analysis for genomic medicine. To this end, we survey six areas where eQTL analyses can provide insight into genetic regulation relevant to health and disease, then conclude with a discussion of the prospects for incorporation of eQTL analysis into translational personalized medicine.

## Definitions and key concepts

**Fig. 1:**
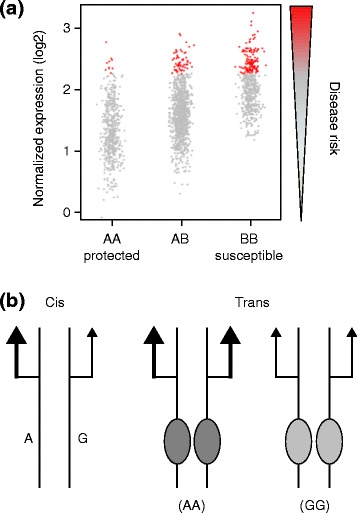
Schematic of eQTLs. **a** eQTLs are defined as sites in the genome where one of the alleles at a single nucleotide polymorphism (SNP) or copy number variation (CNV) is associated with greater average transcript abundance. Relative to disease risk, the allele that increases expression (here *A*) may be associated with protection (as here) or increased susceptibility (*B*). Note that there will always be some number of individuals with the susceptibility or risk genotype whose expression is well within the normal range or even tending in the opposite direction. This consideration suggests that gene expression may be a better indicator of risk than genotype alone, if it can be measured in the right tissue under the right conditions. **b** *Cis*-eQTLs are regulatory polymorphisms that influence transcription of a nearby gene on the same chromosome. Heterozygotes are expected to show allele-specific expression, since one of the alleles, in this case *A*, leads to increased transcription relative to the other (*G*). In general it is assumed that *cis*-eQTLs have differential affinities for transcription factors that bind to promoter, enhancer or repressor elements located within 250 kb on either side of the transcription start site. *Trans*-eQTLs, on the other hand, are polymorphisms at another locus, which increase or decrease expression at both alleles to a similar extent

In humans, most eQTLs are mapped by GWASs using genotyping arrays to measure the genotypes of up to several million single nucleotide polymorphisms (SNPs), and either microarrays or RNA-seq [6] to measure transcript abundance [7]. Statistical association between each SNP and each transcript is computed, revealing places in the genome where there is a linear change in average transcript abundance with each copy of one of the alleles (Fig. 1a). Imputation can be used to increase the search space for possible causal variants, sometimes identifying more statistically significant associations, but more importantly expanding the list of possible causal variants in the vicinity of the initial tagging SNP [8]. If the eQTL polymorphism is located within the vicinity of the transcript, it is called a local eQTL, and the straightforward interpretation is that it (or one or more other variants in linkage disequilibrium with it) directly regulates expression of the gene. As discussed by Albert and Kruglyak in their recent review [5], if the effect is mediated by influencing the binding of a transcription factor, which in turn affects the activity of the RNA polymerase complex on the same physical chromosome, formally the local eQTL effect acts in *cis* [9]. Consequently, the abundance of the transcript derived from that chromosome is altered, which gives rise also to a difference in the overall expression level of the gene. By contrast, distal eQTLs act at a distance through an intermediary, presumably affecting both chromosomes equivalently, and hence are also called *trans-*eQTLs (Fig. 1b). For practical purposes, whether or not an eQTL acts in *cis* or in *trans* is usually defined simply by a distance metric — perhaps the requirement that the polymorphism lies within say 250 kb of the transcription start site of the affected transcript, although local regulation can also extend over 1 Mb [10] and some authors adopt this longer criterion. Conversely, some local eQTLs may influence transcription from both chromosomes to the same degree, effectively acting in *trans*, so a more accurate definition of *cis* and *trans* effects depends on the mechanism of action.

The adoption of RNA-seq has led to the ability to discriminate between variants that influence overall transcript abundance, transcript isoform abundance, allele-specific expression, and novel transcripts including long noncoding RNAs (lncRNAs) [11, 12]. Although the default assumption is that eQTLs regulate the initiation of transcription, variants in the 3’ untranslated region may also regulate steady-state abundance at the level of transcript stability [13]. Others, termed protein abundance QTLs, generally located toward the 5’ end of the transcript, may regulate ribosome binding and hence translation and protein levels without necessarily affecting transcript abundance [14]. Alternative splicing can lead to altered isoform abundance that may not be apparent from microarray analyses that rely on probing of common exons, or RNA-seq analysis pipelines that only survey gene-level transcription. Results from a recent large study of lymphoblast cell lines from the HapMap collection [11] actually indicated that there is considerably more variability at the levels of splicing and usage of alternative transcription start or termination sites than overall abundance. Since many of these differences may be due to relatively rare variants that affect just a few percent of individuals, it is difficult to quantify relative impacts. An important question requiring more focused analysis is to what extent ethnic differences in overall and isoform-specific transcription are due to local eQTLs [15].

Whereas GWASs of disease or clinical phenotypes generally require samples from thousands, if not tens of thousands, of individuals to explain just a minority of the genetic variance seen in a population [16], eQTL analysis has the distinct advantage that significant results can be obtained with as few as 100 samples. Obviously power increases with sample size, and accordingly the number of genes with identified local eQTLs rose from around 5 % for 100 peripheral blood samples to approaching 50 % after meta-analysis of several thousand samples [17]. This reflects increased resolution from variants explaining at least 10 % of the transcript variance, to associations explaining less than 1 %. These effect sizes are large relative to clinical traits, presumably because the effect on binding of transcription or splicing factors is direct. It is worth keeping in mind that the relevant expression may be in a tissue other than the one being profiled, so many associations will be missed because the correct cell type has not been profiled. *Trans*-eQTLs typically explain an order of magnitude less variance than *cis*-eQTLs, and the vast majority of *trans* effects remain unmapped, even though it is estimated that between two thirds and three quarters of the genetic component of gene expression in blood [18] and adipose tissue [19] is due to *trans*-acting factors. Several hundred *trans*-eQTLs have now been identified from meta-analyses [17, 20]; most influence just a handful of transcripts, and collectively they barely make a dent in explaining the overall heritability of gene expression. That is to say, if the average transcript has a heritability of between 20 % and 50 %, and one quarter of this is explained by one or two common local eQTLs, most of the genetic variance remains unexplained.

Furthermore, a hallmark of transcriptomes is a high degree of transcriptional covariance — rather than 10,000 independently transcribed genes per cell type, modules of co-expression exist that influence hundreds or even thousands of transcripts [21, 22]. In peripheral blood, for example, seven major conserved variance components explain well over half the variance of all transcripts [23], reflecting coordinated regulation of genes in the predominate cell types as well as the strength of immune signaling activity, although we do not yet know the genetic basis for this.

## Resources

One of the most important aspects of eQTL analyses for clinical studies is that the data and results are very often made available in an easily accessible format. Although the underlying gene expression data are almost always deposited in one of the major public databases (Gene Expression Omnibus (GEO) or ArrayExpress) [24, 25], there is no such convention for the genotypes or the eQTL results. However, these can generally be accessed either from the journal or individual investigators’ websites, or by request. As the field transitions to whole-genome sequencing in place of genotyping, in an effort to identify rare-variant eQTLs, data access issues may become more problematic, necessitating a reliance on repositories that provide summary statistics [26].

Happily, there are several existing resources (highlighted in Table 1) that facilitate browsing by any investigators whether or not they have expertise in the underlying statistical methods. The GeneVar database [27] at the Sanger Institute, for example, presents results from 726 HapMap lymphocyte cell lines and three different tissues (lymphocyte cell line (LCL), adipose, and skin) from 856 healthy female twins enrolled in the MuTHER study, in each case searchable either by transcript or eQTL SNP. Manhattan plots visualize the significance of all of the associations across a locus, while scatter plots visualize the variance in transcript abundance for each genotype at a single SNP. More recent HapMap Project [28] results generated from whole-genome sequencing and comprehensive RNA-seq are presented in a bespoke genome browser at the Geuvadis resource [11]. For investigators interested in peripheral blood eQTLs, the Blood eQTL browser [17] provides a meta-analysis of *cis*- and *trans*-eQTL results from dozens of international studies in table format, searchable by gene or SNP. Approximately half of all genes return no results, while a handful return hundreds of results at extremely small false discovery rate levels, reflecting high linkage disequilibrium (but note that the database does not yet include imputed SNPs). An emerging critical resource is the genotype tissue expression (GTEx) Portal [29, 30], which will allow users to query a database of eQTLs detected in multiple tissues — initially 13 tissues from at least 60 postmortem donors but it is expected to include more than 20 tissues in 900 donors by the end of 2015. The NCBI is developing a searchable browser for this project that allows users to select a tissue, apply filters, and download reported eQTLs. Two other very useful resources that integrate eQTLs with other types of genomic and clinical association data are the Gilad and Pritchard laboratory resources hosted by the University of Chicago, and the Pickrell laboratory GWAS browser hosted by the New York Genome Center [31].

**Table 1:** Some prominent eQTL resources

Resource	URL	Nature of data
GeneVar	http://www.sanger.ac.uk/resources/software/genevar/	eQTL visualization tools
Geuvadis	http://www.ebi.ac.uk/Tools/geuvadis-das/	HapMap LCL eQTLs
Blood eQTL	http://genenetwork.nl/bloodeqtlbrowser/	Blood eQTL meta-analysis
GTEx Portal	http://www.gtexportal.org/home/	Multi-tissue eQTL study
NCBI	http://www.ncbi.nlm.nih.gov/projects/gap/eqtl/index.cgi	Searchable database of GTEx
Chicago eQTL	http://eqtl.uchicago.edu/Home.html	eQTLs with genomic features
Pickrell laboratory	http://gwas-browser.nygenome.org/	eQTLs with GWAS association

*eQTLs* expression quantitative trait loci, *GTEx* genotype tissue expression, *GWAS* genome-wide association study, *LCL* lymphocyte cell line

Once eQTLs are recognized to fall into extended haplotype blocks in which hundreds of SNPs may be responsible for the associations detected by GWASs, the issue of fine-mapping the causal variant arises. For many clinical purposes, this may not be important, but it is also becoming clear that local eQTLs can operate over hundreds of kilobases, affecting a gene or genes that are not the most proximal to the causal SNP [9, 11]. In addition, statistical arguments strongly support the inference that in many cases there may be multiple independently acting SNPs responsible for the eQTL effect at each locus [32–34]. Although there is no consistency yet to the usage of the term eSNP (which is commonly used interchangeably with eQTL), we suggest that fine-mapped variants that are candidate causal mediators of the eQTL observation should be called eSNPs.

Unfortunately, there is as yet no database for such eSNPs, perhaps because there is only good functional evidence for a small number of individual sites. In the meantime, there are several resources that can help investigators narrow down the pool of candidate eSNPs within an eQTL region. RegulomeDB [35], for example (http://regulomedb.org/), ranks SNPs according to whether there are functional data from a variety of ENCODE assays. Type 1 SNPs have a known eQTL association as well as evidence from DNase I hypersensitive sites (DHSs), chromatin immunoprecipitation, predicted transcription factor binding, or reporter gene assays. HaploReg [36] serves a similar purpose. Several related measures exist, including ones such as CADD that integrate sequence conservation into the functional inference [37, 38], and these have been shown to provide useful prioritization of candidate variants for disease [39]. There is as yet little consistency to the use of these scores. Another immediate need is high-throughput functional assays to experimentally validate that individual sites do impact gene expression [40], and eventually to establish whether and how multiple variants at a single locus function together. Table 2 provides a list of some prominent recent eQTL studies in four domains: interaction effects, integration with epigenetics, technical advances, and eQTLs for response to perturbation.

**Table 2:** Some prominent recent eQTL publications

Reference	Topic
Interaction effects	
[108]	Comprehensive two-locus interaction screen for epistatic eQTL effects
[109]	Debate surrounding epistatic interactions described in [108]
[110]	Interaction effects influencing allele-specific gene expression
[111]	QTLs influencing the variance of gene expression
[49]	Estimation of architecture of variance from pedigree studies
Chromatin and epigenetics	
[112]	Genetic and epigenetic regulation of lncRNA expression
[113]	Role of histone modification and transcription factor binding on eQTL effects
[114]	Identification of genetic variants influencing histone modification
[115]	Role of methylation QTLs in modifying eQTL effects
[116]	Contributions of methylation and expression QTLs in fibroblasts
Technical advances	
[11]	eQTL identification through RNA-seq plus whole-genome sequencing
[117]	Joint eQTL and protein expression analysis
[118]	eQTLs in ten regions of the human brain
Disease studies	
[93]	eQTLs for the immune response to tuberculosis
[94]	eQTLs in childhood malaria and parasitemia
[95]	Changes in blood eQTL profile associated with myocardial infarction
[119]	eQTLs in COPD
[80]	*Cis*-regulatory influences on gene expression in colorectal cancer
Perturbation studies and response eQTLs	
[84]	Conditional dependence of eQTLs in monocytes
[85]	Conditional dependence of eQTLs in lymphocytes
[86]	Conditional dependence of eQTLs in dendritic cells
[15]	Monocyte- and lymphocyte-specific eQTLs across ethnicities

*COPD* chronic obstructive pulmonary disease, *eQTL* expression quantitative trait locus, *lncRNA* long noncoding RNA, *RNA-seq* RNA-sequencing

## Six uses for eQTL analysis in genome medicine

We turn now to the question of how eQTLs can be used in the service of genomic medicine. As with other measures derived from GWASs, the primary utility is indirect, namely improved understanding of disease mechanisms. Applications in personalized medicine, whether diagnostic, predictive, or therapeutic, lie in the future, but we conclude the review with a discussion of the notion of transcriptional risk scores.

### Identifying which gene corresponds to a GWAS disease or trait association

Undoubtedly the most direct application of eQTL analysis is in fine-mapping a GWAS association to a specific gene within the interval. Given the linkage disequilibrium structure in the human genome, the resolution of GWASs is typically to haplotype blocks that may cover anywhere from 20 kb to upwards of 100 kb. Cross-ethnicity comparisons may improve the resolution [15, 41, 42], but even in the theoretical limit where just a single SNP is shown to cause the peak association, it cannot be concluded that the SNP acts on the nearest gene. This is true of the situation both when the GWAS SNP lies in a gene desert (where no known transcripts have been identified) or lies in a high-gene-density region. Since over three quarters of GWAS hits appear not to be associated with potentially deleterious protein-coding variants [1, 43], the vast majority are likely to be regulatory. eQTL analysis provides an effective solution for quickly ascertaining which gene in a region of association is most likely dysregulated in the disease. Note that variants in the gene need not even be in linkage disequilibrium with the eSNP.

A textbook example of this application is provided by the hypercholesterolemia association identified at chromosomal interval 1p13.3, where any one of seven genes could plausibly be responsible for one of the largest known genetic effects on serum cholesterol levels [44]. eQTL analysis in liver biopsies demonstrated that the abundance of two transcripts, *PSRC1* and *SORT1*, tends to be highest in homozygotes for the minor allele, with heterozygotes having an intermediate abundance. Subsequently, substitution of the minor for the major variant affecting a C/EBP transcription factor binding site was shown to reduce expression from a luciferase reporter gene, confirming the identity of rs12740374 as the eSNP. Most importantly, both knockdown and increase of *Sort1*, but not *Psrc1*, in mouse had the predicted effects on elevating and reducing serum cholesterol levels, respectively [44]. Thus, eQTL profiling reduced the set of candidate genes that needed to be assayed to establish the identity of the causal gene — and thereby to define a novel drug target, the Golgi transmembrane receptor SORT1.

Most studies do not go to such experimental depths to prove the identity of the causal gene that is regulated by a GWAS SNP. The literature is full of inferential statements based simply on the observation that a high percentage of disease associations localize to an eQTL. This is a somewhat risky business, since the concordance of two correlations — an SNP with gene expression and with disease risk — does not establish causation and instead could be due to pleiotropy. Nevertheless, there is little doubt that peak associations are enriched for local eQTL effects, and algorithms such as Regulatory Trait Concordance [45] have been developed to provide statistical support for the argument that the causal variant for a particular eQTL is the same as a causal variant for a GWAS hit. The aforementioned Geuvadis study [11] also showed that the likelihood that a GWAS SNP is an eQTL declines with its rank in a linkage disequilibrium block: the peak association need not be the causal one, but eQTL results are consistent with near-peak ones being the functional variant in many cases. Similarly, the Coloc package in R weighs evidence that an SNP associates with two or more traits, which can include a disease and transcript [46]. Further statistical method development is expected to lead to substantial improvements in fine-mapping causal regulatory variants, and may illuminate novel mechanisms that do not necessarily require disruption of transcription factor binding [34].

### Defining the cell types or regulators most likely involved in the etiology of a disease

The pathophysiology of most common diseases is often restricted to a limited number of tissue/cell types or organ systems [47, 48]. Therefore, if the majority of the genetic susceptibility for disease acts through gene regulation, it is likely that tissue-specific eQTLs underlie some disease risk. Owing mainly to accessibility, the majority of our knowledge about eQTLs comes from expression levels measured in either whole blood [49–51] or isolated cellular components of blood [52, 53]. However, results from eQTL and whole-genome studies in multiple tissues have demonstrated that the genetic control of gene regulation often differs between tissues [18, 54]. Overlap of eQTLs among tissues has been one approach used to address the question of the degree of common genetic control between tissues [19, 55, 56]. Most such studies have independently mapped eQTLs in two or more tissues and contrasted the number of eQTLs found in both tissues, arriving at estimates of the overlap between a pair of tissues ranging from 12 to 80 %. These studies are important in that they identify loci that have a common effect on transcripts between tissues. However, there are clear limitations that restrict the conclusions that can be drawn.

A principal limitation is the inability to detect eQTLs that have small effect sizes, especially given the severe multiple testing burden that is inherent in eQTL mapping. Alternative approaches that jointly weight the probability of eQTLs across multiple tissues have been demonstrated to increase power to detect multi-tissue eQTLs [57]. Numerous other analytical issues influence our ability to interpret comparative eQTL studies, including technical issues related to data quality and experimental design, differences in the way that statistical models are formulated, and variable sample sizes. Of course, true heterogeneity of effects across tissues is also certainly present.

An alternative way to address the question of cross-tissue concordance is to ask what is the total amount of genetic variance of transcript levels shared between tissues? Such estimates, called cross-tissue heritability [18] and genetic correlation analysis [49], use experimental designs that include related individuals to estimate the total amount of genetic variance that is shared between two tissues. The resulting estimates are similar to heritability estimates in that they represent the sum of all genetic effects, irrespective of their identification in an eQTL analysis, and have suggested that, on average, the total amount of genetic control shared between tissues is low.

The tissue specificity of the genetic control of transcripts leads to an interesting challenge when investigating the functional role of genome-wide association (GWA) loci through eQTL interpretation in cases where the pathophysiology of the disease stems from a different set of tissues to the eQTL data [58]. A common limitation in disease genomics studies is the difficulty of obtaining pathologically relevant tissue on which to measure expression. In such situations we would caution against the over-interpretation of the mechanism by which GWA causal loci influence disease susceptibility, unless there is specific knowledge on shared genetic control of the transcript levels between the relevant tissues. To understand the mechanisms of disease susceptibility and to develop preventative and targeted therapies, we ultimately require knowledge of genetic control of regulatory variation in many different tissues [3, 48, 55]. Projects such as GTEx [29] will provide an invaluable tool for identifying eQTLs that are conserved across tissues and cell types and will ultimately allow knowledge gained from expression levels measured in more readily available tissues to be better utilized.

Despite these limitations, various strategies are used to infer, given a list of SNPs, which biological processes they have in common and/or to generate an interaction network that implicates a particular biochemical or cellular process. These include text-mining (tools such as GRAIL [59]), protein–protein interaction networks (DAPPLE, STRING [60, 61]), and gene set enrichment analysis [62]. One that is somewhat specific to eQTL analysis is cell-type enrichment. The simplest strategy is to ask in which cell type is the set of transcripts affected by multiple different eQTLs most strongly expressed, and whether there is a bias toward co-expression in a particular cell type. Thus, for the approximately 100 known inflammatory bowel disease-associated loci, more than half of which have eQTLs in blood, expression is enriched in several immune cell types, notably T cells, dendritic cells, and NK cells, but not B cells or neutrophils [63]. This does not prove that those cell types contribute to the etiology (for example, elevated expression in a low-abundance cell type could be pathological), but it does seem intuitive that if multiple eSNPs act in the same cell type, then that cell type is likely to contribute to the disease or trait associated with the same SNPs. Another example is the apparent enrichment of schizophrenia-associated SNPs in the vicinity of genes expressed in monocytes, and hence likely to impact derived macrophages and inflammation [64]. Actually, this approach does not require that the SNPs are shown to be eQTLs, it only requires that the transcript is enriched in abundance in the cell type, but we envisage that as projects such as GTEx expand the range of eQTL tissues, actual eQTL signature enrichment will generate more robust inferences.

Indirect incorporation of eQTLs into the enrichment assessment was first reported by Maurano and colleagues [65], who drew inferences concerning cell type specificity from DHS data. They started with the observation that regulatory variants are enriched in DHSs, and asked whether the inclusion of increasing numbers of low-significance GWAS associations resulted in enrichment for DHSs in specific cell types that have been extensively characterized as part of the ENCODE project [66]. Positive results were observed for interleukin (IL)-17-secreting T cells in samples from patients with Crohn’s disease, confirming immunological experiments, and for fetal cardiomyocytes in patients with cardiac QT interval. Just as interestingly, DHSs were relatively depleted from neuronal cell types with respect to association with multiple sclerosis, strongly arguing against a neuronal role in the pathology of this autoimmune brain disease. In a parallel analysis, the authors asked whether there was enrichment for predicted transcription-factor-binding sites in the DHSs associated with autoimmune diseases, malignancies, or neuropsychiatric disorders. They found 22 transcription factors with binding sites in at least eight DHSs that are located in established GWAS loci, and generated an immune regulatory network involving STAT1, STAT3, NF-κB and PPAR-γ that is highly likely to mediate the aberrant expression associated with disease [65]. Different networks were implicated in the other two disease categories.

### Highlighting likely causal genes among differentially expressed genes

A relatively underappreciated application of eQTL analysis is that it may facilitate scans of differentially expressed genes for causal loci. As noted above, it is common in transcriptome studies to observe that hundreds of genes are co-expressed [22, 23]. Consequently, when investigators contrast normal and diseased tissue, they typically identify a large number of transcripts that are either induced or suppressed in the cases compared to controls [3, 67]. It is difficult to know which of these genes contribute to the pathology of a disease, and which are ‘going along for the ride’ owing to co-expression. For example, comparison of peripheral blood from healthy controls and Crohn’s disease patients reveals several hundred transcripts that are differentially expressed [68], but only a fraction of these are associated with the disease by GWASs (despite the majority of the GWAS loci being eQTLs). eQTL analysis suggests a strategy for prioritizing the causal genes among the differentially expressed ones, on the assumption that only the co-expressed genes that also harbor a disease association are causally involved.

We agree with the systems genomics perspective that the intersection between differential expression, eQTLs, and GWAS disease associations has the highest probability of highlighting genes most likely to contribute to pathology [69, 70]. The advantage of eQTL analysis in this context is that it does not require the large sample sizes that disease GWASs require. Hence, lower-significance SNPs can be scanned for eQTL effects, and instead of asking whether the transcripts are enriched in a cell type, it is possible to ask whether they are enriched in the differentially expressed genes in patient samples. To date, the vast majority of eQTL studies have been performed on healthy controls, and only a handful of studies have compared eQTLs in cases and controls. The GTex project [30] is a very welcome development, expanding the number of tissues available for eQTL analysis to include most sites of pathology (for example, liver, kidney, ovary, testes, skin, various brain regions), but it is unlikely to include large numbers of patients. However, tissue biopsies from patients are often feasible and should be prioritized. Another possible approach will be the differentiation of induced pluripotent stem cells from cases and controls [71], although there is no guarantee that this will generate expression profiles that mirror the pathological state in patients.

### Localizing potential drivers and modifiers of cancer

The Cancer Genome Atlas projects have been at the forefront of integrative genomic approaches to disease by generating datasets that combine whole-exome sequencing with RNA-seq from matched tumor–normal tissue pairs [72–74]. Most of the emphasis in the field has been on the detection of genes that are significantly mutated in cancer, the so-called drivers that harbor deleterious somatic mutations more often than expected by chance [75, 76]. In parallel, GWASs have revealed that most cancers also have a common variant susceptibility profile that includes regulatory variants [77]. Aberrant methylation is also well recognized as a risk factor for some classes of tumor, which indirectly implicates altered transcription [78, 79]. Thus, while the search for druggable targets has focused on aberrant protein sequences, there is increasing recognition that altered gene and protein expression is an important component of oncogenesis. Quite possibly, gene expression signatures may emerge as predictors of therapeutic response to drug or immune therapy.

Recent studies of colorectal cancer have highlighted two novel directions for eQTL analysis [80–82]. The first is quantification of aberrant gene expression in tumor relative to control tissue for the purpose of identifying novel drivers. Just as an excess of somatic protein-coding mutations in a subset of genes marks them as likely cancer promoting genes, so too an excess of somatic regulatory mutations in theory should mark genes whose loss or gain of activity contributes to tumor growth. The analytical problem is that we do not as yet have high-confidence tools for defining which somatic mutations affect regulatory DNA, and the technical problem is that it takes whole-genome, not just exome, sequencing, to find novel mutations. To overcome these issues, Ongen and colleagues [80] searched for transcripts that displayed allelic dysregulation in colorectal tumors relative to matched normal colon — that is, genes for which the ratio of transcript abundance from the two chromosomes had significantly changed. Whether due to point mutations, loss of heterozygosity, or even mutated *trans*-regulators, the existence of an average of 200 events in each of 103 tumor pairs allowed the authors to identify 71 potential regulatory drivers, 9 of which overlap with suspected drivers from protein-coding mutations. Functional validation experiments will be required to establish that the expression level of each gene does drive or modulate cancer progression.

The second novel direction is the discovery of cryptic eQTLs, which are regulatory polymorphisms whose activity is condition-dependent [80, 83]. In the environment of the tumor, altered expression of critical transcription or splicing factors renders regulatory polymorphisms that are silent in normal tissue functional. This possibility follows from the interpretation that *cis*-eQTLs act through altered affinity of the two alleles for a regulatory protein. In the absence of that protein, there is no functional difference, but when the nuclear environment changes the polymorphism now influences gene expression. This can occur in a positive or negative fashion, and may affect genes that are or are not actively transcribed in normal tissue. The research strategy here is simply to compare eQTL profiles between normal and tumor tissue and test for a significant interaction effect. Again, given the relatively large effect sizes of eQTLs, it turns out that there is sufficient statistical power to find condition-dependent effects even with samples as small as 100 pairs. Furthermore, scans for transcription-factor-binding sites found enrichment for six proteins, IRX3, E2F4, NFIL3, TFAP2A, CUX1 and LEF1, each of which was indeed altered in abundance in the cancer biopsies [80]. Similar analyses of other cancer types are eagerly awaited, as are efforts to assess whether this type of analysis can have translational importance in the context of personalized cancer treatment.

### Mechanistic dissection of regulatory switches

Extending the concept of condition dependence, a series of studies have begun to analyze how immune activation affects gene expression regulation by explicitly perturbing primary cells in culture and then contrasting eQTL profiles with baseline [84–86]. A similar approach has been applied in model organisms, giving rise to “response eQTLs”, namely loci that are associated with the response to perturbation [87]. The context is that eQTL analysis of accessible tissues is intrinsically limited if the effects that are clinically relevant are highly condition-specific. Immunologists, for example, have emphasized that the properties of myeloid and lymphoid cells are very different between circulating peripheral blood and sites of infection or inflammation, that cellular age is relevant to function, and that stimulation of immune cells elicits transcriptional responses. To this end, eQTL profiling of distinct cell types such as monocytes, T cells, and dendritic cells does identify eQTLs that are cell-type specific and not recovered in peripheral blood mixtures (and lymphoblast cell lines appear to be quite different again). Cell-type-specific eQTLs might also be recovered by including measured cell-type abundance as a covariate in the regression model used to identify associations in tissues that are a mixture of cells [88], such as blood. It should be emphasized that the majority of eQTLs observed in leukocytes are consistent across cell types [53], but it is possible that the 10–20 % that have opposite effects in, or are only observed in, one cell type are the most critical for individual pathology.

The ImmGen consortium recently published two papers examining the impact of ex vivo activation on dendritic cells and T lymphocytes [85, 86], and Fairfax and colleagues [84] published a similarly impressive study of monocytes. The three critical messages of these studies are: (1) that activation state does alter eQTL profiles quite substantially, with half of all naïve-state eQTLs disappearing upon stimulation and at least as many only observed in stimulated cells; (2) there is a temporal dynamic to the response profiles as cells move through their regulatory state switch; and (3) several key shifts involve key immune mediators that have been repeatedly identified in GWASs for inflammatory and autoimmune diseases. For example, Lee and colleagues [86] differentiated peripheral blood monocytes into dendritic cells from 534 healthy donors, and then stimulated them with lipopolysaccharide, influenza virus, or interferon-β. They detected 121 response eQTLs from a targeted analysis of 415 immunity-related genes, 57 of which were common to all three treatments and 38 implicated in inflammatory or autoimmune disease. One of these, affecting the expression of interferon response factor 7 (*IRF7*) in *cis*, was in turn a *trans*-eQTL for seven other genes after influenza virus stimulation. Targeted replacement of another SNP that binds the interferon-response transcription factor STAT2 by CRISPR/Cas9 abrogated the stimulus response of the eQTL *SLFN5*. Clearly the clever combination of eQTL analysis with experimental perturbation has great potential to illuminate the genetic basis of individual immunological responses.

These results raise the question, taken up again in the final section of this review, of whether eQTL profiling may be useful in translational medicine. An underappreciated aspect of genetic prediction is that no matter the specificity and sensitivity of genotypic risk scores (GRSs), predictive utility will always be constrained by the heritability and prevalence of the disease. If the heritability is less than 50 %, which is the case for most diseases, the best genetics can do is classify individuals into risk categories. If the disease is rare, often in the vicinity of 1 % prevalence there will always be a high ratio of unaffected to affected individuals who have GRSs in the upper quartiles or deciles [89, 90]. Consequently, there is considerable interest in using gene expression as a biomarker for disease prediction, and/or to help classify disease subtypes among affected individuals. The expression levels of genes that are regulated by eQTLs that are also associated with disease would seem a priori to be strong candidate biomarkers. The context-dependence of eQTL effects suggests, however, that the expression profiling will need to be performed either in situ, at the site of pathology, or ex vivo under conditions that mimic pathogenesis.

### Exploration of genotype-by-environment interactions

It follows, to the extent that environment modifies disease risk, that gene expression should also be evaluated in the context of the environment within which individuals live. Given the global epidemiological transition towards diseases that are prevalent in the Western world, arguably the most relevant environmental parameter is lifestyle. To this end, Idaghdour and colleagues surveyed gene expression in peripheral blood from desert nomads, urban slum dwellers, and rural villagers in southern Morocco [91]; they observed pervasive differentiation among these populations independent of ethnicity, involving at least a third of the transcriptome. Similar results were observed on a smaller scale for Indian villagers relative to residents of Suva in Fiji [92]. However, eQTL analysis of the Moroccan sample was conspicuous for the complete absence of genotype-by-environment interactions (G × E) involving common regulatory polymorphisms: at each one of approximately 400 eQTLs, the sign and magnitude of effect was the same for rural villagers and city residents despite significant main effects of population [91]. This result implies that *cis*-eQTL effects are quite robust to lifestyle changes. As pointed out in the paper, it nevertheless suggests a mechanism for G × E at the level of high-order phenotypes, since individuals beyond thresholds of high or low expression will tend to be those with the combination of the relevant homozygous genotype living in the environment in which expression tends to be greater or lesser in general.

Another type of environmental factor that could modify eQTL effects is disease status, as described for cancer above. Active tuberculosis, for example, impacts the expression of thousands of genes, and induces condition-dependent eQTL effects for key modulators of immune signaling [93]. High parasitemia also pervasively alters peripheral blood gene expression, likely via signaling between infected red blood cells and lymphocytes, and, correspondingly, eQTLs are affected [94]. A third example is atherosclerosis, as individuals at high risk of myocardial infarction have dozens of modulated eQTL effects [95], again in peripheral blood — although to date these have not been linked to variants that associate with myocardial infarction. More studies contrasting healthy and diseased individuals, in a diversity of tissues, are needed before we can draw general conclusions regarding how disease modifies eQTL profiles. One interpretation is that the changes are merely in response to pathology, but the more compelling possibility is that possession of specific eQTL profiles results in altered transcriptome states that are themselves pathological.

A third important cellular environmental factor is that afforded by genetic population structure, namely differences in allele frequencies among populations. Several of the first eQTL studies established that in LCLs at least 15 % of transcripts are differentially expressed among the three major population groups represented in the HapMap samples (Yoruban Africans, Caucasians, and Han or Japanese East Asians), and, correspondingly, population-specific local eQTLs were identified [96, 97]. Similar results are found using peripheral blood, driven either by differential abundance of cell types such as neutrophils and T helper 17 cells, or by differential effects of eQTLs within cells [98]. Owing to allele frequency differences among populations, statistical power to detect eQTLs can vary, so the identification of population-specific effect sizes requires a significant interaction effect. The aforementioned ImmGen Consortium has demonstrated that at least 30 % of eQTLs in lymphocytes fail to replicate across all three population groups, but the proportion of local eQTLs that truly have different effect sizes is likely to be somewhat less [15]. Of particular clinical relevance will be establishing to what degree effects differ upon stimulation or in diseased tissues, and, subsequently, whether any differences have population-biased therapeutic implications.

## eQTLs in translational medicine

In this review, we have discussed various ways in which eQTL analysis is impacting genome medicine from the perspective of understanding mechanisms of disease. We conclude with some thoughts on whether eQTLs may also be of more translational importance. As with findings derived from GWASs and whole-exome sequencing, there are at least three opportunities for translation: precision medicine, prediction, and nosology.

Precision medicine refers to efforts to identify the proximate genetic cause of a disease or condition in an individual patient [99, 100]. It has quickly gained attention through the rapid introduction of next-generation sequencing approaches in the domains of cancer and pediatric congenital abnormalities, where the objective is to identify one or a few rare mutations that may explain the pathology. Even though it is doubtful that most variants are fully penetrant and thus sufficient to explain causality completely, the overwhelming evidence is that in more than a quarter of cases, whole-exome sequencing can identify necessary deleterious variants [101, 102]. Many believe that rare regulatory ‘causal’ variants will also be identified once the switch to whole-genome sequencing is made and algorithmic detection of regulatory defects improves. Such variants will by definition be rare local eQTLs. Their definitive identification will be aided by high-throughput methods for establishing a functional impact on transcript abundance.

Genetic prediction refers to efforts to establish relative risks for individuals based on the sum of their genotypic risks [103]. Most often it assumes a GRS, but here we introduce the concept of a transcriptional risk score (TRS). This is the sum of standardized gene expression measures for transcripts influenced by eQTLs for a disease, measured where possible in the relevant tissue. It is not the same as a predictor based on quantitative trait transcripts [104, 105], which are simply transcripts found to be associated with a trait. Rather, it is asking whether a joint measure of transcript abundance due to GWAS associations is a better predictor of the trait or disease than an allelic sum. For inflammatory or autoimmune disorders, for example, GWASs have identified upwards of 100 risk loci, the majority of which are eQTLs [63]. We can polarize gene expression relative to risk by assessing whether the high-risk genotype is associated with increased or decreased transcript abundance, and then sum the polarized *z*-scores to generate a TRS, which will be correlated with the GRS.

**Fig. 2:**
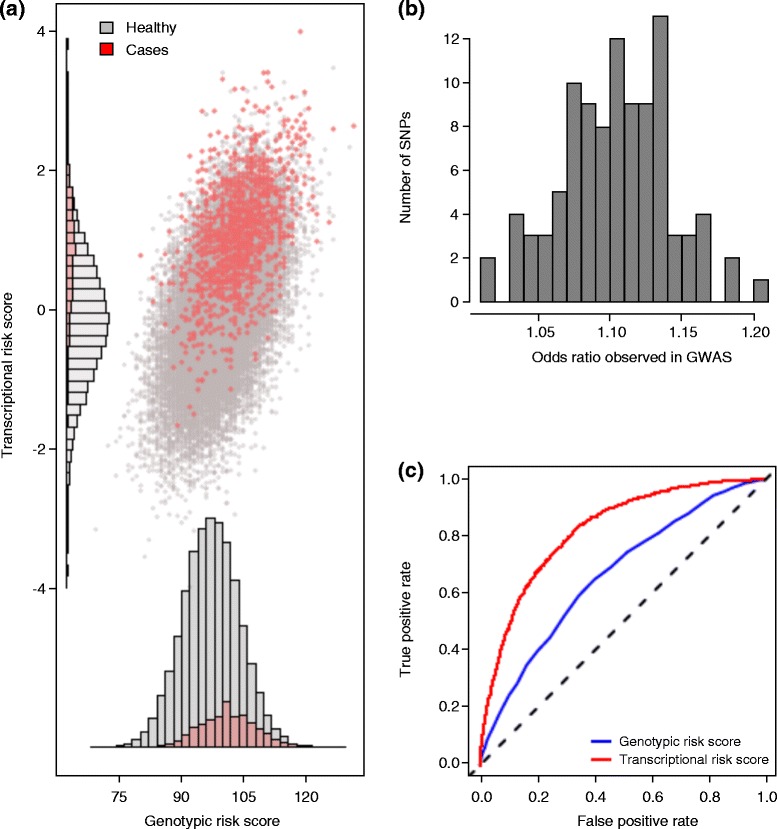
Transcriptional and genotypic risk scores. **a** The relationship between the allelic sum genotypic risk score (GRS) and the polarized sum of transcriptional risk score (TRS) *z*-scores in a simulation of 100,000 individuals in whom disease is observed in the individuals in the highest decile of an underlying phenotype with 50 % heritability. The correlation between GRS and TRS is highly significant, but red points highlight how the individuals with the highest risk for disease can differ with respect to genotypic and transcriptional risk at eQTL loci. **b** Frequency distribution of inferred genotype effect sizes for the 100 genes, median 1.09-fold risk, all but one less than 1.2-fold risk, indicating compatibility with an infinitesimal model of complex disease genetics. **c** Receiver operating curves for the TRS and GRS, showing that the TRS under this model achieves much higher true positive rates (sensitivity) for smaller false positive rates (higher specificity). *GWAS* genome-wide association study, *SNP* single nucleotide polymorphism

To illustrate this concept, we performed a simulation study assuming that disease incidence is affected by the expression of 100 genes, each regulated by a single eQTL that explains 25 % of its variance yet is associated with a less than 1.2-fold increase in disease susceptibility. Collectively these eQTL explain one half of the risk. Figure 2a illustrates how different individuals will be inferred to be in the highest risk category for the allelic sum GRS and the TRS estimated in 100,000 people with a disease prevalence of 10 %. Since the eQTL genotypes act through transcript abundance, we might expect the TRS to be a better predictor than the GRS, at least under conditions in which the transcriptional effects are additive. This is indeed the case, as the area under the receiver operating curve for the TRS is significantly greater than the corresponding GRS (Fig. 2c shows a typical iteration). There are many different classes of model that can explain the relationship between gene expression and disease, leading to different types of TRS, including weighting of the eQTL effect size, only considering extreme expression values, and incorporating the structure of the affected pathway into the analysis. Unfortunately, we were not able to identify sufficiently large eQTL disease studies to test the proposition that TRSs have greater predictive utility than GRSs.

Finally, eQTLs have considerable potential for nosology. Whereas GWASs typically make no attempt to sub-classify individuals with respect to genotypic risk, there are signs that once large numbers of loci have been identified it may be fruitful to consider the nature of the risk variants that each individual possesses in order to better understand individualized sources of risk. For example, type 2 diabetes risk alleles can be divided into those that more strongly affect insulin production (homeostatic model assessment (HOMA)-B) or insulin resistance (HOMA-IR), and it follows that individuals with SNP profiles biased in either direction may have different subtypes of diabetes [106]. Similarly, rheumatoid arthritis variants affect genes that can be placed in pathways that respond differently to various drugs, and it is plausible that treatment might be targeted in an individualized manner on the basis of enrichment of variants linked to specific drugs [107]. This notion is readily generalized to the supposition that individuals who share combinations of eQTLs may exhibit particular symptomology and/or respond to specific treatments. Ideally, it would not be necessary to actually measure gene expression in the patient if eQTLs defined in an independent disease cohort prove to be strongly predictive enough to classify individuals by genotype alone.

## Conclusion

These considerations lead us toward a model for personalized medicine in which genotype and transcript abundance are utilized in an integrative manner. In some cases, a single eQTL may be sufficient to highlight a critical risk factor or pharmacogenetic target, in others it may be a cumulative eQTL risk score, and of course in others gene expression may prove to be either unnecessary or uninformative. The field is currently benefiting from the incorporation of ENCODE data for the purposes of refining eQTLs to causal eSNPs, and from the development of tools for meta-analysis that are greatly increasing resolution. Open source databases are ensuring that the results of studies are widely accessible, and we expect that pooling of resources will also facilitate mega-analyses that provide opportunities for deeper statistical inference. Once these approaches are extended to diverse tissues through projects such as GTEx, and eventually to comparisons of diseased and normal tissues, eQTLs are set to become a core component of personalized medicine.

## Abbreviations

CNV: Copy number variation
COPD: Chronic obstructive pulmonary disease
DHS: DNase I hypersensitive site
eQTL: Expression quantitative trait locus
G × E: Genotype-by-environment interaction
GRS: Genotypic risk score
GTEx: Genotype tissue expression
GWA: Genome-wide association
GWAS: Genome-wide association study
HOMA: Homeostatic model assessment
lncRNA: Long noncoding RNA
RNA-seq: RNA-sequencing
SNPs: Single nucleotide polymorphisms
TRS: Transcriptional risk score
